# Stacking Polymorphism in PtSe_2_ Drastically Affects Its Electromechanical Properties

**DOI:** 10.1002/advs.202201272

**Published:** 2022-06-02

**Authors:** Roman Kempt, Sebastian Lukas, Oliver Hartwig, Maximilian Prechtl, Agnieszka Kuc, Thomas Brumme, Sha Li, Daniel Neumaier, Max C. Lemme, Georg S. Duesberg, Thomas Heine

**Affiliations:** ^1^ Chair of Theoretical Chemistry Technische Universität Dresden Bergstrasse 66 Dresden 01069 Germany; ^2^ Chair of Electronic Devices RWTH Aachen University Otto‐Blumenthal‐Str. 2 Aachen 52074 Germany; ^3^ Insitute of Physics Faculty of Electrical Engineering and Information Technology (EIT 2) Universität der Bundeswehr München Werner‐Heisenberg‐Weg 39 Neubiberg 85577 Germany; ^4^ Helmholtz‐Zentrum Dresden‐Rossendorf Permoserstrasse 15 Leipzig 04318 Germany; ^5^ AMO GmbH Advanced Microelectronic Center Aachen Otto‐Blumenthal‐Str. 25 Aachen 52074 Germany; ^6^ Chair of Smart Sensor Systems Bergische Universität Wuppertal Lise‐Meitner‐Str. 13 Wuppertal 42119 Germany; ^7^ Chair of Electronic Devices RWTH Aachen University Otto‐Blumenthal‐Str. 2 Aachen 52074 Germany; ^8^ Department of Chemistry Yonsei University Seodaemun‐gu Seoul 120‐749 Republic of Korea

**Keywords:** density‐functional theory, piezoresistive sensors, PtSe_2_, Raman characterization, stacking disorder, two‐dimensional materials

## Abstract

PtSe_2_ is one of the most promising materials for the next generation of piezoresistive sensors. However, the large‐scale synthesis of homogeneous thin films with reproducible electromechanical properties is challenging due to polycrystallinity. It is shown that stacking phases other than the 1T phase become thermodynamically available at elevated temperatures that are common during synthesis. It is shown that these phases can make up a significant fraction in a polycrystalline thin film and discuss methods to characterize them, including their Seebeck coefficients. Lastly, their gauge factors, which vary strongly and heavily impact the performance of a nanoelectromechanical device are estimated.

## Graphical abstract

Order matters: In this study, the authors show that stacking disorder occurs at temperatures between 400 and 500 °C in PtSe_2_, which is a candidate material for next‐generation piezoresistive sensors. However, some stacking phases might be beneficial due to their higher gauge factors, albeit their characterization is challenging.

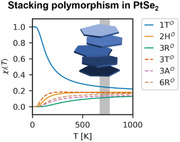

## 1 Introduction

Two‐dimensional (2D) materials are excellent candidates for next‐generation nanoelectromechanical devices.^[^ ^1^ ^]^ They feature high in‐plane stiffness and strength but are easily bend,^[^ ^2^ ^]^ for example as suspended membranes spanned over a cavity.^[^ ^1^ ^]^ Their electrical response can be tailored with their thickness,^[^ ^3^, ^4^, ^5^ ^]^ including metal‐to‐semiconductor transitions for a reduced number of layers.^[^ ^6^, ^7^ ^]^ Especially, the noble‐metal dichalcogenides (NMDCs), such as PtSe_2_,^[^ ^8^ ^]^ excel in piezoresistive sensors due to their high gauge factors,^[^ ^9^, ^10^ ^]^ long‐term stability at ambient conditions,^[^ ^10^, ^11^ ^]^ and low‐temperature synthesis.^[^ ^12^ ^]^ Furthermore, they have successfully been applied in optical devices, such as phototransistors^[^ ^13^, ^14^ ^]^ and photodetectors.^[^ ^11^, ^15^, ^16^, ^17^ ^]^

For integrated devices, chemical vapor deposition (CVD) and thermally assisted conversion (TAC) are the preferred options to obtain large‐scale thin films of high‐quality NMDCs with controllable thicknesses.^[^ ^1^, ^10^, ^18^ ^]^ PtSe_2_ is especially promising in this regard because of the low temperature of 400 °C needed for TAC, which is compatible with complementary metal‐oxide semiconductor (CMOS) integration.^[^ ^1^, ^9^, ^12^, ^19^ ^]^ In both cases, challenges arise due to the polycrystallinity of such‐obtained films (including polymorphism^[^ ^20^, ^21^ ^]^ and random crystallite alignment^[^ ^22^ ^]^), the role of the substrate, as well as contacting them in the CMOS integration process.^[^ ^1^ ^]^ The TAC process has the advantage that additional substrate transfer steps may be avoided by area‐selective growth.^[^ ^23^ ^]^ At every step, a non‐invasive and timely characterization of the films is required, which is typically carried out using Raman spectroscopy.^[^ ^20^ ^]^

Previously, we performed an extensive study of different NMDC polytypes with respect to the metal coordination, e.g., trigonal prismatic coordination in the MoS_2_‐type 2H phase and octahedral coordination in the CdI_2_‐type 1T phase.^[^ ^24^ ^]^ In this nomenclature, the number indicates the number of layers per unit cell and the letter abbreviates the crystal system.^[^ ^25^ ^]^ PtSe_2_ has been observed in different coordination phases: For instance, Wang et al.^[^ ^26^ ^]^ showed the formation of MoS_2_‐like 2H‐PtSe_2_ nanoflakes by chemical vapor deposition at 900 °C, while Tong et al.^[^ ^27^ ^]^ reported that single‐layer of the same material was formed by liquid immersion of Pt(111) in Na_2_Se. Furthermore, Lin et al.^[^ ^21^ ^]^ observed 1H/1T in‐plane PtSe_2_ heterostructures at different annealing temperatures, while Xu et al.^[^ ^28^ ^]^ obtained trigonal prismatic bilayer PtSe_2_.

The octahedrally coordinated 1T phase of PtSe_2_ is regarded as the most stable phase.^[^ ^29^ ^]^ It attracted great attention, because it is semimetallic in the bulk, but becomes semiconducting for fewer layers.^[^ ^6^, ^7^, ^30^ ^]^ However, other stacking phases of the 1T phase have been investigated to much less extent. To clearly distinguish the metal environments, in the following, we introduce the superscript *O* for octahedral coordination and the superscript *T* for trigonal prismatic coordination. A figure illustrating this naming scheme is given in Figure S1 (Supporting Information). All stacking phases in this study feature octahedral coordination.

A rhombohedral stacking phase with three layers per unit cell (3R*^O^*‐PtSe_2_) has been observed as a minor side phase by O'Brien et al.^[^ ^20^ ^]^ and has been studied by Villaos et al.^[^ ^31^ ^]^ using DFT calculations. They conclude that it is close in energy to the 1T*^O^* phase at 0 k and semiconducting in the bulk. Since other stacking orders of the 1T*^O^* phase appear closer in energy than other coordination phases, they can grow competitively at elevated temperatures in TAC and CVD processes, contributing to the formation of polycrystalline thin films. Importantly, the symmetry reduction from the high‐symmetry 1T*^O^* phase to lower‐symmetry stacking phases can lead to semiconducting properties. This has a significant impact on the electronic characteristics of polycrystalline thin films: For example, other stacking phases might help to explain the large discrepancy of electronic mobilities of PtSe_2_ found in literature, ranging from lower than 1 ^[^ ^19^, ^32^, ^33^ ^]^ to 625 cm^−2^ V^−1^ s^−1^.^[^ ^22^ ^]^

Here, we present an intensive study of the stacking phases of layered PtSe_2_ using density‐functional theory (DFT) to characterize their role in the formation of polycrystalline films in the temperature range between 0 and 1000 K. Notably, we find that lower‐symmetry stacking orders than the previously reported AA‐stacking order in 1T*^O^*‐PtSe_2_^[^ ^20^, ^29^ ^]^ can make up a significant fraction at elevated temperatures. We confirm the formation of the 3R*^O^* phase reported by Villaos et al.^[^ ^31^ ^]^ at experimental temperatures, as well as four other metastable stacking phases (2H*^O^*, 3T*^O^*, 6R*^O^*, and 3A*^O^*, where A stands for anorthic to avoid confusion between trigonal and triclinic). These have significant impact on the electronic properties of PtSe_2_ thin films, including semiconducting behavior, large gauge factors, and anisotropic conductivities. We show that the stacking phases cannot be easily distinguished by their Raman signatures and would require high‐resolution transmission electron microscopy (HRTEM) analysis. We show that the correlation to thermoelectric properties, such as the Seebeck coefficient, may yield a good indication of the presence of other stacking phases.

## 2 Results and Discussion

### 2.1 Structures, Stabilities, and Characterization

**Figure 1:**
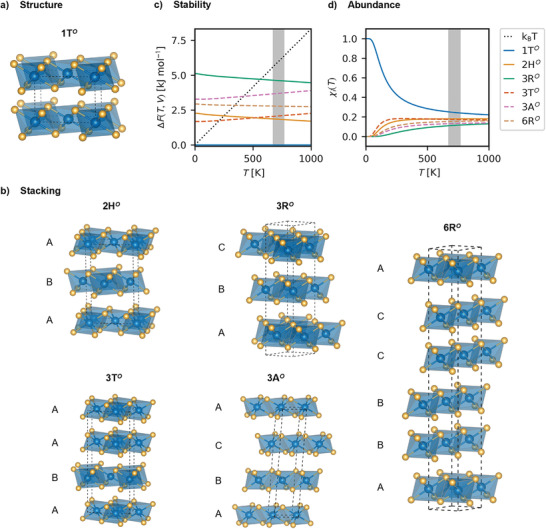
a) Most common 1T*^O^* structure of PtSe_2_. b) The five additional stacking phases studied in this work, including their label, and stacking description (additional data can be found in the Supporting Information). c) Relative thermodynamic stability of all six stacking phases based on the Free Helmholtz energy at constant volume. d) Relative thermodynamic abundance based on the partition function at different temperatures. The gray stripe indicates experimental synthesis temperatures.

We arrive at six thermodynamically likely stacking phases (1T*^O^*, 2H*^O^*, 3R*^O^*, 3T*^O^*, 6R*^O^*, 3A*^O^*) shown in **Figure** **1**a,b by sampling above 200 possible stacking orders (details in the Supporting Information). They are summarized in **Table** **1**.

**Table 1:** Nomenclature of the six stacking orders obtained in this work, their electronic band gaps (Δ) of bulk forms (calculated at HSE06+SOC level), their average interlayer distances (*d*), and estimated “in‐plane” gauge factors (GF) and “randomly aligned” gauge factors (GF*) at the PBE level

Label	Stacking	Crystal family	Space group	Δ [eV]	*d* [Å]	GF	GF*
1T*^O^*	AA	Trigonal	$P\bar{3}m1$	0.00	4.957	6 to 10	1 to 4
2H*^O^*	AB	Hexagonal	P 6_3_ *mc*	0.00	5.385	−1 to 12	−4 to 7
3R*^O^*	ABC	Rhombohedral	$R\bar{3}m$	0.63	5.703	−43 to 16	−34 to 10
3T*^O^*	AAB	Trigonal	P 3*m*1	0.00	5.242	5 to 9	−24 to 12
3A*^O^*	ABC	Anorthic^a)^	P 1	0.44	5.496	−4 to 22	−4 to 3
6R*^O^*	AABBCC	Rhombohedral	$R\bar{3}m$	0.00	5.326	−346 to −63	−370 to −92

^a)^ We label this stacking order as anorthic instead of triclinic to avoid confusion with trigonal 1T*^O^*.

**Figure 2:**
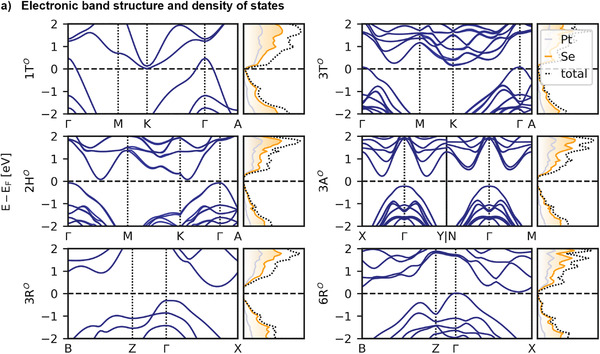
a) Electronic band structure and density of states of the six bulk stacking phases at the HSE06 level of theory including spin–orbit coupling (SOC) for a subsection of the Brillouin Zone. The full path along the Brillouin Zone can be found in the Supporting Information.

All six stacking orders are locally stable showing no imaginary phonon modes (see Figure S2, Supporting Information). The 1T*^O^*, 2H*^O^*, 3T*^O^*, and 6R*^O^* stacking phases are bulk semimetals (see **Figure** **2**a and Figure S3, Supporting Information), while 3R*^O^* and 3A*^O^* are semiconductors. The 3R*^O^* and 3A*^O^* stacking phases have much larger mean interlayer distances than the 1T*^O^* phase (see Figures S4 and S5, Supporting Information). This may indicate that a high‐pressure synthesis favors the formation of the 1T*^O^* phase with smaller interlayer distance. In the 3T*^O^*, 3A*^O^*, and 6R*^O^* phases, the interlayer distance disproportionates between different stacking regions (see Figure S5, Supporting Information). Importantly, regions with the same stacking (e.g., AA, BB, CC) always feature the smallest interlayer distance (4.957 to 5.032 Å). Differently stacked regions feature interlayer distances between 5.385 to 5.703 Å. This may lead to additional X‐ray reflections. For comparison, we show the simulated powder X‐ray diffraction patterns in Figure S6 (Supporting Information).

At synthesis temperatures of 400–500 °C,^[^ ^10^ ^]^ all six stackings become thermodynamically available with their Free Helmholtz Energy differences being lower than *k*_B_ *T* (see Figure 1c). Whereas the high‐symmetry 1T*^O^* and 3R*^O^* stacking phases feature no low‐lying optical branches in their phonon spectra, the other four phases have states available at excitation energies below 50 cm^−1^ due to the coupling of the acoustic modes of different layers (see Figure S2, Supporting Information). Thus, the lower‐symmetry phases become favored by entropy at elevated temperatures. The 1T*^O^* phase is still the most stable one in the temperature range between 0 and 1000 K, but its relative abundance in equilibrium decreases to about 30% (see Figure 1c). The relative abundance shows that at experimental temperatures, all six stacking phases can be present in thin films at relatively evenly distributed fractions, with the 1T*^O^* phase being in the majority. The estimation of the abundance does not take external pressure, substrate effects and kinetic effects into account, which may be used to tune the chemical equilibrium in favor of one stacking phase or another. Another important factor is the film thickness, where thin films cannot feature all stacking orders (see Figure S7, Supporting Information).

**Figure 3:**
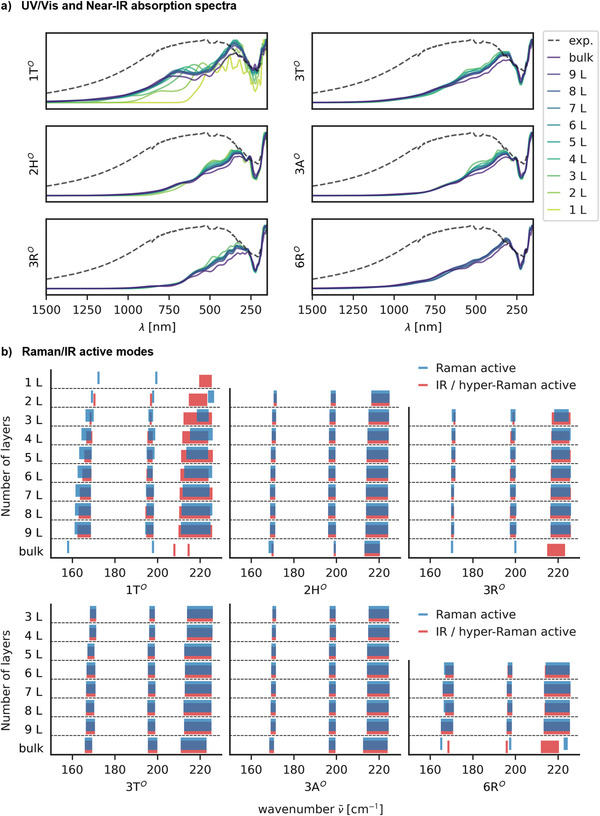
a) Calculated absorption spectrum based on the imaginary part of the dielectric function for different layer numbers of the six stacking phases vs. experimental absorption spectrum for a thickness of PtSe_2_ of 15 nm. b) Frequencies of the Raman‐ and IR‐active modes of the six stacking phases for different layer numbers and bulk. The width of the bar indicates the lower and upper range of the respective modes.

Most stackings feature semimetallic characteristics in the bulk (see Figure 2a), which should dominate the conductance if grain size and film thickness are sufficiently large and the fraction of the 3R*^O^* and 3A*^O^* stacking phases is small. These semimetals also feature broad absorption tails ranging into the near‐infrared regime (see **Figure** **3**a), with the 1T*^O^* phase being most prominent. For thin films with a reduced layer number, the semimetallic stacking orders undergo a metal‐to‐semiconductor transition, whereas the semiconducting phases broaden their band gaps (see Figure S8, Supporting Information). Consequently, their absorption shifts further into the visible regime. This effect is most pronounced for the 1T*^O^* phase, whereas the absorption of the other stackings is affected to much less extent. The experimental absorption spectrum of a 15 nm thick film of PtSe_2_ is shown in Figure 3a for comparison. Due to the 1T*^O^* phase being the most abundant, we argue that it dominates the absorbance in a polycrystalline thin film. Hence, the absorption spectrum does not help to distinguish different stacking phases.

Likewise, we conclude that distinguishing stackings by Raman and IR spectroscopy is difficult (see Figure 3b). The calculated E_g_ mode of the 1T*^O^* phase is strongly affected by interlayer coupling, shifting from 158.1 to 172.3 cm^−1^ from bulk to monolayer, respectively (exp.: 174 cm^−1^ for 3 to 5 layers).^[^ ^20^ ^]^ On the other hand, the A_1g_ mode is less affected by interlayer coupling and shifts very little (exp.: 205 cm^−1^).^[^ ^20^, ^34^ ^]^ Experimentally, shifts of about 10 cm^−1^ have been observed for the E_g_ mode of PtSe_2_ depending on the sample thickness.^[^ ^20^ ^]^ For thin films, the interlayer coupling of the E_g_ modes gives rise to a distribution of frequencies, which narrows to a single frequency for thick samples due to stacking rigidity. In Figure 3b, we indicate the upper and lower bound of these distributions for each group of modes (e.g., E_g_, A_1g_, E_u_, and A_1u_ in the case of the 1T*^O^* phase) for up to nine layers, which corresponds to roughly 4.3 nm thickness. The A_1u_ and E_u_ modes are not Raman‐active in bulk and monolayer due to higher symmetry but become symmetry‐allowed for few layers, however, they are usually not observed in experiments.^[^ ^20^ ^]^ For all six stacking phases, their Raman/IR‐active modes feature smaller frequency distributions due to weaker interlayer coupling and fall into the variance of the active modes of the 1T*^O^* phase. Furthermore, Gulo et al.^[^ ^34^ ^]^ have shown a large temperature‐dependence of the Raman frequencies due to anharmonicity, which can vary by 4 to 6 cm^–1^ at 500 K. Hence, in a polycrystalline mixture, the measurement of Raman shifts alone does not give conclusive evidence of other stacking phases.

### 2.2 Electronic, Thermoelectric, and Mechanical Properties

For nanoelectromechanical applications, the reproducible synthesis of PtSe_2_ thin films with a large piezoresistive effect is desirable. Polycrystallinity is disadvantageous since the appearance of grain boundaries leads to worse mechanical stability and hinders electrical conductivity. However, it is unclear which stacking phase of PtSe_2_ is the most desirable one for nanoelectromechanical systems. Film thicknesses between 3 and 20 nm of PtSe_2_ are expected to be semimetallic experimentally,^[^ ^6^, ^18^, ^30^ ^]^ which is beneficial for low contact resistances. On the other hand, semiconductors are expected to have higher gauge factors, such as mono‐ and bilayer 1T*^O^* PtSe_2_,^[^ ^6^ ^]^ as well as the 3R*^O^* and 3A*^O^* stacking phases, and thin films of the 2H*^O^*, 3T*^O^* and 6R*^O^* phases (see Figure S8, Supporting Information).

The relevant quantity for the gauge factor (GF) is the change of the resistivity *ρ* under strain *ε*:^[^ ^1^ ^]^

(1) $$\mathrm{GF}=\frac{\Delta R/R}{\Delta L/L}=\frac{\Delta R/R}{\varepsilon }=1+2\nu +\frac{\Delta \rho /\rho }{\varepsilon }$$

Here, *R* is the resistance, *L* is the length and *ν* is the Poisson's ratio. We outline an approach to estimate the GF in the Supporting Information, where we distinguish between two possible scenarios of polycrystallinity that are likely for layered structures. In the first case, the samples may feature vertical stacking disorder, but are generally well‐aligned in the xyplane. Then, the biggest contribution to the sample resistivity comes from the inplane elements of the resistivity tensor and the GF mainly depends on these elements. In the second case, the samples might be randomly aligned with sufficiently large grain sizes, which has been observed in PtSe_2_ for film thicknesses bigger than 40 to 50 nm.^[^ ^22^ ^]^ Then, we employ Hill's definition^[^ ^35^ ^]^ for the Poisson's ratio of a polycrystalline aggregate

(2) $$\nu^{*}=\frac{1}{2}\left(1-\frac{3G_{V}}{3K_{V}+G_{V}}\right)$$

with *G*_V_ being a mean Voigt shear modulus and *K*_V_ being a mean Voigt bulk modulus. In the second case, we average over the trace of the resistivity tensor.

**Figure 4:**
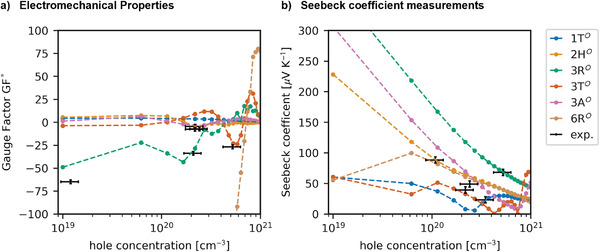
a) Experimental gauge factors vs. theoretical estimate of pure phases. b) Experimental Seebeck coefficients vs. theoretical estimate. Bulk carrier concentrations were estimated including an error coming from the film thickness varying between different measurements taken from Lukas et al.^[^ ^33^ ^]^ and Prechtl et al.^[^ ^23^ ^]^ and gauge factors were taken from Lukas et al.^[^ ^33^ ^]^

Since the resistivity is a function of both temperature and carrier concentration due to intrinsic doping, so is the GF (see **Figure** **4**a). In Table 1, we summarize both definitions of the GF at 300 K with the range coming from experimentally observed carrier concentrations. This shows that the carrier concentration must be controlled carefully in experiment, because it may heavily affect the sign and magnitude of the GF. Interestingly, we predict small GFs for the 1T*^O^*, 2H*^O^*, 3T*^O^*_,_ and 3A*^O^* stacking phases, whereas the 3R*^O^* and 6R*^O^* stacking phases feature the largest negative values (up to −370). Hence, these might actually be the most desirable phases for piezoresistive sensing if they can be grown as a majority fraction. Furthermore, the discrepancy between the “in‐plane” scenario and the “randomly aligned” scenario indicates that polycrystallinity may also have a huge impact, with a tendency to worsen the GF. The calculated GFs agree well with experimentally observed GFs (see Figure 4a), but do not allow for a distinction of different stacking phases.

This leaves the question how the stacking phases might be assigned in experiment. We argue that a correlation of different techniques to HRTEM or XRD analysis might yield conclusive evidence of the stacking phases, similar to a finger print.^[^ ^33^ ^]^ Together with electromechanical measurements, the Seebeck coefficient might be a useful indicator for sample quality. We estimate the Seebeck coefficient for a temperature gradient from 300 to 400 K as function of the carrier concentration for different stacking phases (see Figure 4b with method details in the Supporting Information). The semimetallic stacking phases, such as the 1T*^O^* phase, tend to feature smaller Seebeck coefficients. For small carrier concentrations, the Seebeck coefficient is largest with the 3R*^O^* phase showing the highest Seebeck coefficient for all stacking phases. The estimated Seebeck coefficients agree well with the experimentally observed range but do not allow a clear distinction of stacking phases on their own. We propose that a high Seebeck coefficient might indicate a large fraction of the 3R*^O^* phase, which could be beneficial for nanoelectromechanical sensing due to its higher GF. On the other hand, we must point out as well that the Seebeck coefficient is affected by other experimental factors, such as the grain size and grain boundaries, which we cannot consider in our theoretical model yet.

## 3 Conclusion

We investigated stacking polymorphism in PtSe_2_ and found six thermodynamically relevant phases at experimental synthesis temperatures of 400–500 °C. We show that these cannot be distinguished by Raman/IR‐spectroscopy or their UV/vis absorption. When estimating their electromechanical properties, we notice a huge impact of the stacking order and alignment on the gauge factors: Some stacking orders become semiconducting and, thus, lead to higher gauge factors. We highlight the Seebeck coefficient as a potential indicator of the 3R*^O^* phase. We argue that a clear distinction of stacking phases requires a correlative analysis of different measurement techniques and HRTEM or XRD. Such a correlative picture is important to judge the sample quality for piezoresistive sensing, where stacking orders other than the 1T*^O^* phase might actually be beneficial.

## 4 Experimental Section

A detailed explanation of the methods can be found in the Supporting Information. All calculations were carried using the *Fritz‐Haber‐Institute ab‐intitio materials simulations package* (FHI‐aims).^[^ ^36^ ^]^ The sampling of structures was performed employing the Atomic Simulation Environment (ASE)^[^ ^37^ ^]^ and the Space Group library (spglib).^[^ ^38^ ^]^ Phonons and harmonic energies were calculated using FHI‐vibes^[^ ^39^ ^]^ and phonopy.^[^ ^40^ ^]^ Resistivities and Seebeck coefficients were extracted via Boltzmann Transport Theory as implemented in BoltzTraP2.^[^ ^41^ ^]^ PtSe_2_ films were fabricated from sputtered or evaporated platinum (Pt) layers by means of thermally assisted conversion (TAC) as published earlier.^[^ ^20^, ^42^ ^]^ Measurements of the gauge factors and Seebeck coefficients were undertaken as published earlier.^[^ ^33^ ^]^

### Statistical Analysis

The theoretical predictions were obtained from ab initio calculations. Experimental errors of the charge carrier concentration, Gauge Factors and Seebeck coefficients were estimated from Lukas et al.^[^ ^33^ ^]^

## Conflict of Interest

The authors declare no conflict of interest.

## Supporting information

Supporting Information

## Data Availability

The data that support the findings of this study are available from the corresponding author upon reasonable request.
