# Supplementary material for: Stacking Polymorphism in PtSe2 Drastically Affects Its Electromechanical Properties
Source: Adv Sci (Weinh). 2022 Jun 2;9(22):2201272. doi: 10.1002/advs.202201272 (PMC9353474; doi:10.1002/advs.202201272)
Supplement: Supplementary file 1 — Supporting Information [file ADVS-9-2201272-s001.pdf]

ADVANCED SCIENCE
Open Access

Supporting Information

for *Adv. Sci.*, DOI 10.1002/advs.202201272

Stacking Polymorphism in $PtSe_2$ Drastically Affects Its Electromechanical Properties

*Roman Kempt, Sebastian Lukas, Oliver Hartwig, Maximilian Prechtl, Agnieszka Kuc, Thomas Brumme, Sha Li, Daniel Neumaier, Max C. Lemme, Georg S. Duesberg and Thomas Heine**

Stacking polymorphism in $PtSe_2$ drastically affects its electromechanical properties

Roman Kempt, Sebastian Lukas, Oliver Hartwig, Maximilian Prechtl, Agnieszka Kuc, Thomas Brumme, Sha Li, Daniel Neumaier, Max C. Lemme, Georg S. Duesberg, Thomas Heine**

Roman Kempt, Dr. Thomas Brumme, Prof. Dr. Thomas Heine
Chair of Theoretical Chemistry, Technische Universität Dresden
Bergstrasse 66, 01069 Dresden, Germany
E-mail: thomas.heine@tu-dresden.de

Dr. Agnieszka Kuc, Prof. Dr. Thomas Heine
Helmholtz-Zentrum Dresden-Rossendorf
Permoserstrasse 15, 04318 Leipzig, Germany

Prof. Dr. Thomas Heine
Department of Chemistry
Yonsei University, Seodaemun-gu, Seoul 120-749, Republic of Korea

Sebastian Lukas
Chair of Electronic Devices, RWTH Aachen University, Otto-Blumenthal-Str. 2, 52074 Aachen, Germany

Dr. Sha Li
AMO GmbH, Advanced Microelectronic Center Aachen, Otto-Blumenthal-Str. 25, 52074 Aachen, Germany

Prof. Dr. Daniel Neumaier
Chair of Smart Sensor Systems, Bergische Universität Wuppertal, Lise-Meitner-Str. 13, 42119 Wuppertal, Germany
AMO GmbH, Advanced Microelectronic Center Aachen, Otto-Blumenthal-Str. 25, 52074 Aachen, Germany

Prof. Dr. Max C. Lemme
Chair of Electronic Devices, RWTH Aachen University, Otto-Blumenthal-Str. 2, 52074 Aachen, Germany
AMO GmbH, Advanced Microelectronic Center Aachen, Otto-Blumenthal-Str. 25, 52074 Aachen, Germany

Oliver Hartwig, Maximilian Prechtl, Prof. Dr. Georg S. Duesberg
Insitute of Physics, Faculty of Electrical Engineering and Information Technology (EIT 2), Universität der Bundeswehr München, Werner-Heisenberg-Weg 39, 85577 Neubiberg, Germany

Keywords: $PtSe_2$, two-dimensional materials, piezoresistive sensor, Raman characterization, stacking disorder, density-functional theory

Supporting Information

1. Method details

1.1. Sampling of stacking phases

We generated stacking-disordered phases of $PtSe_2$ based on a single-layer of $1T^{O}$-$PtSe_2$ by allowing for all possible combinations of five translations and three rotations in up to three layers in a bulk unit cell, resulting in 225 combinations. The allowed translations in fractional coordinates were (0.0 **a** + 0.0 **b**), (0.5 **a** + 0.0 **b**), (1/3 **a** + 1/3 **b**), (1/3 **a** + 2/3 **b**) and (0.5 **a** + 0.5 **b**). The rotation angles considered were 0°, 30° and 60°, which preserve the trigonal lattice. This pool of initial structures was reduced by symmetry analysis as implemented in the Atomic Simulation Environment (ASE)[1] and the Space Group Library (spglib)[2]. The structure (atomic positions and cell parameters) was optimized within a 2 × 2 × 1 supercell to allow for spontaneous symmetry reduction. All calculations were performed using FHI-aims[3] on tight tier 1 numeric atom-centered orbitals employing the PBE functional[4] with added non-local many-body dispersion correction (MBDnl)[5] on Monkhorst-Pack Γ-centered k-grids with k-point densities of 12 points per Å till the final geometries were converged up to threshold of $5 \cdot 10^{-3}$ eV per Å via FHI-vibes.[6]

1.2. Calculation of phonons and thermodynamic stability

For structures remaining from the sampling above, phonons were calculated using phonopy[7] in converged super cells depending on the initial size and shape of the primitive unit (supercell matrices: 2H, 3T, 3A: 2 × 2 × 2; 1T, 6R: 3 × 3 × 3, 3R: [[3,-3,0],[2,2,-4],[1,1,1]]). Structures that exhibited imaginary frequencies were subsequently discarded for being unstable. We calculated the thermodynamic stability with the Free Helmholtz energy: $\Delta F(T,V) = ZPE + F_{phon}(T,V) + E_{elec}(0\ \mathrm{K})$, where the electronic energy was taken from DFT at the PBE level including the MBDnl energy. The Raman/IR-active Γ-point frequencies were extracted with the Bilbao crystallographic server.[8]

1.3. Calculation of relative abundances

We estimate relative abundances at experimental temperatures from the partition functions of the bulk solids. Formally, the phononic part of the partition function of a bulk solid is defined as[9]

$$Z_{phon} = \prod_{\mathbf{k},\nu} \frac{e^{-h\nu(\mathbf{k})\cdot 2\beta}}{1 - e^{-h\nu(\mathbf{k})\cdot\beta}}$$

with β being the Boltzmann factor. However, the calculation of Z_{phon} is impractical because it diverges at the Γ-point due to the acoustic modes. Still, the Free Helmholtz energy at constant volume can be obtained from the natural logarithm of Z_{phon}:

$$F_{phon}(T,V) = -\beta \cdot \ln\left(Z_{phon}\right)$$

This expression can be simplified by replacing the summations in $\ln\left(Z_{phon}\right)$ with an integral over the phonon density of states $g(\nu)$:[6,9]

$$F_{phon}(T,V) \approx \int g(\nu)\left(\frac{h\nu}{2} + \beta \cdot \ln\left(1 - e^{-h\nu\cdot\beta}\right)\right)\mathrm{d}\nu$$

This approach is numerically stable because of the vanishing density of states of the acoustic modes at Γ for low frequencies. We use this to estimate Z_{phon}:

$$Z_{phon} \approx e^{-F_{phon}(T,V)\cdot\beta}$$

The total partition function is then estimated as $Z_{phon} \cdot Z_{elec}$ and the relative thermodynamic abundance at equilibrium for infinite time is calculated assuming non-interacting phases:

$$\chi_i(T) = \frac{z_{i,phon} z_{i,elec}}{\sum_j z_{j,phon} z_{j,elec}} = \frac{z_{i,phon} \cdot e^{-\Delta E_{i,elec}\cdot\beta}}{\sum_j z_{j,phon} \cdot e^{-\Delta E_{j,elec}\cdot\beta}}$$

1.4. Calculation of electronic and mechanical properties

For the remaining six stacking phases, as well as two-dimensional stacks of these phases for up to nine layers (starting from the smallest number of layers needed to build that stacking order), we calculated the electronic band structures, densities of states, and absorption spectra at the HSE06[10] level including atomistic scalar-relativistic corrections (ZORA) and spin-orbit coupling (SOC) as implemented in FHI-aims on tight tier 1 numeric atom-centered orbitals with k-point line densities of 12 points per Å.[3]

The elastic tensor of the six bulk phases was calculated by numerical forward differentiation of the strained unit cell for a strain of 0.001 Å with subsequent relaxation of atomic positions. The Poisson's ratios were calculated from the compliance tensor as implemented in the matscipy library.[11]

We calculated the Boltzmann conductivity tensor $\boldsymbol{\sigma}/\tau$ as implemented in BoltzTraP2[12] in the constant relaxation time approximation for all bulk stacking phases, as well as for strained systems. We estimate experimental Seebeck coefficients via

$$U \approx -(T_2 - T_1) \cdot \mathbf{Tr}\left(\mathbf{S}(\bar{T})\right) \text{ ,}^{[13]}$$

with $\bar{T}$ being the average temperature $(T_1 + T_2)/2$ and $\mathbf{Tr}(\mathbf{S}) = (S_{xx} + S_{yy} + S_{zz})/3$ being the mean trace of the Seebeck tensor. The voltage U is typically plotted as $|\Delta U|/\Delta T$ and the Seebeck coefficient is extracted from the slope via linear regression.[13]

In the following, we motivate two estimates of the gauge factor assuming either that a) the layers are well-aligned in the xy plane or b) randomly aligned and polycrystalline. In both cases, we assume the same uniform relaxation time τ for the strained and unstrained systems, which allows us to calculate the change of resistivities from the Boltzmann conductivities given relative to an unknown relaxation time:

$$\boldsymbol{\rho}/\tau = \boldsymbol{\sigma}^{-\mathbf{1}}/\tau$$

$$a) \quad GF \approx 1 + \nu_{xy} + \nu_{yx} + \frac{1}{2}\left(\frac{\frac{\rho'_{xx} - \rho_{xx}}{\rho_{xx}}}{\varepsilon_{xx}} + \frac{\frac{\rho'_{yy} - \rho_{yy}}{\rho_{yy}}}{\varepsilon_{yy}}\right)$$

Here, $\boldsymbol{\rho}$ is the Boltzmann resistivity tensor, $\boldsymbol{\sigma}$ the Boltzmann conductivity tensor, ν_{xy} and ν_{yx} are Poisson's ratios, and ε is the strain along a certain direction. In case a) we average over the in-plane elements of the Poisson's ratio and the resistivity tensor.

In case b), we employ the definition of polycrystalline elasticity given by Hill[14] to calculate the Poisson's ratio from the Voigt bulk modulus K_V and Voigt shear modulus G_V:

$$K_V = \frac{(c_{11} + c_{22} + c_{33}) + 2 \cdot (c_{12} + c_{23} + c_{31})}{9}$$

$$G_V = \frac{(c_{11} + c_{22} + c_{33}) - (c_{12} + c_{23} + c_{31}) + 3 \cdot (c_{44} + c_{55} + c_{66})}{15}$$

Where c_{ij} are elements of the compliance tensor $\mathbf{C}$ in Voigt notation. Then, the polycrystalline Poisson's ratio ν^* is given by:

$$\nu^{*} = \frac{1}{2}\left(1 - \frac{3G_V}{3K_V + G_V}\right)$$

And the polycrystalline gauge factor for strain in x-direction GF^* is estimated from the trace average of the resistivity tensor $\rho^* = (\rho_{xx} + \rho_{yy} + \rho_{zz})/3$:

$$b) \quad GF^* \approx 1 + 2 \cdot \nu^* + \left(\frac{\Delta\rho^*}{\rho^*}\right)/\varepsilon_{xx}$$

1.5. Experimental methods

$PtSe_2$ films were fabricated from sputtered or evaporated platinum (Pt) layers by means of thermally assisted conversion (TAC) as published earlier.[15,16] To determine the charge carrier concentrations, the $PtSe_2$ films were transferred from their centimeter-scale SiO_2 or quartz growth substrates onto highly p-doped Si / 90 nm SiO_2 substrates. Six-port Hall devices were defined by optical lithography and reactive ion etching as described in our previous study.[17] Hall measurements were then performed to extract the sheet charge carrier density, as also described in the same study.[17]

For gauge factor (GF) and Seebeck coefficient measurements, the $PtSe_2$ films were transferred onto flexible polyimide substrates and contacted with Nickel (Ni) electrodes, as again described in the same study,[17] including details of the GF measurement using a steel beam set-up. The same samples were used for the measurement of the Seebeck coefficient in a thermoelectric measurement set-up where the two ends of the device were clamped onto hotplates of different temperatures to create a temperature gradient. While one hotplate was kept at room temperature, the other one was heated up to 100 °C gradually. During this process, the voltage between the two Ni contacts of the device was measured using a nanovoltmeter. A linear fit of the recorded voltage versus the temperature difference was used to extract an approximate Seebeck coefficient for the given temperature range.

UV/Vis transmission measurements were performed on a 15 nm $PtSe_2$ film, previously transferred onto a quartz substrate, in a Perkin Elmer LAMBDA 1050 UV/Vis spectrophotometer at wavelengths between 190 nm and 2400 nm. The measurement was normalized using a reference measurement of a plain quartz substrate. The absorption curve was then calculated from the transmission curve with neglected reflectance ($\mathrm{Absorption} = 1 - \mathrm{Transmission}$).

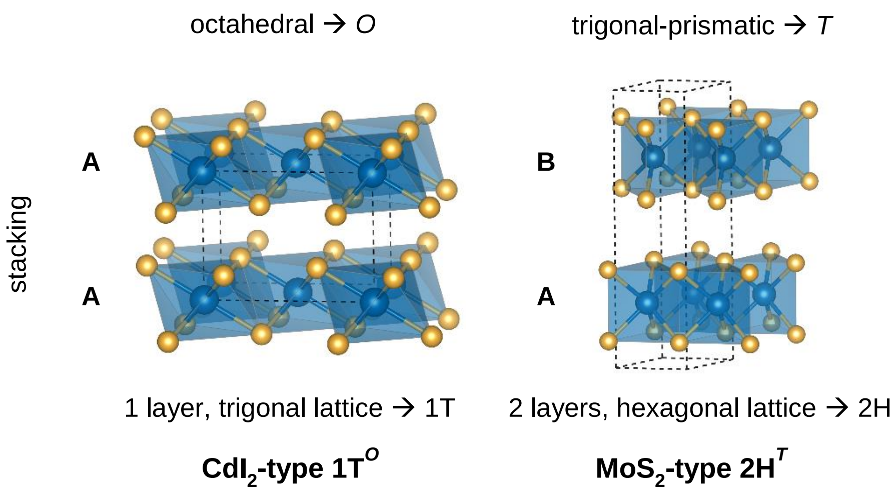

Figure S1 – Short-hand nomenclature in this work to distinguish between common TMDC polytypes.

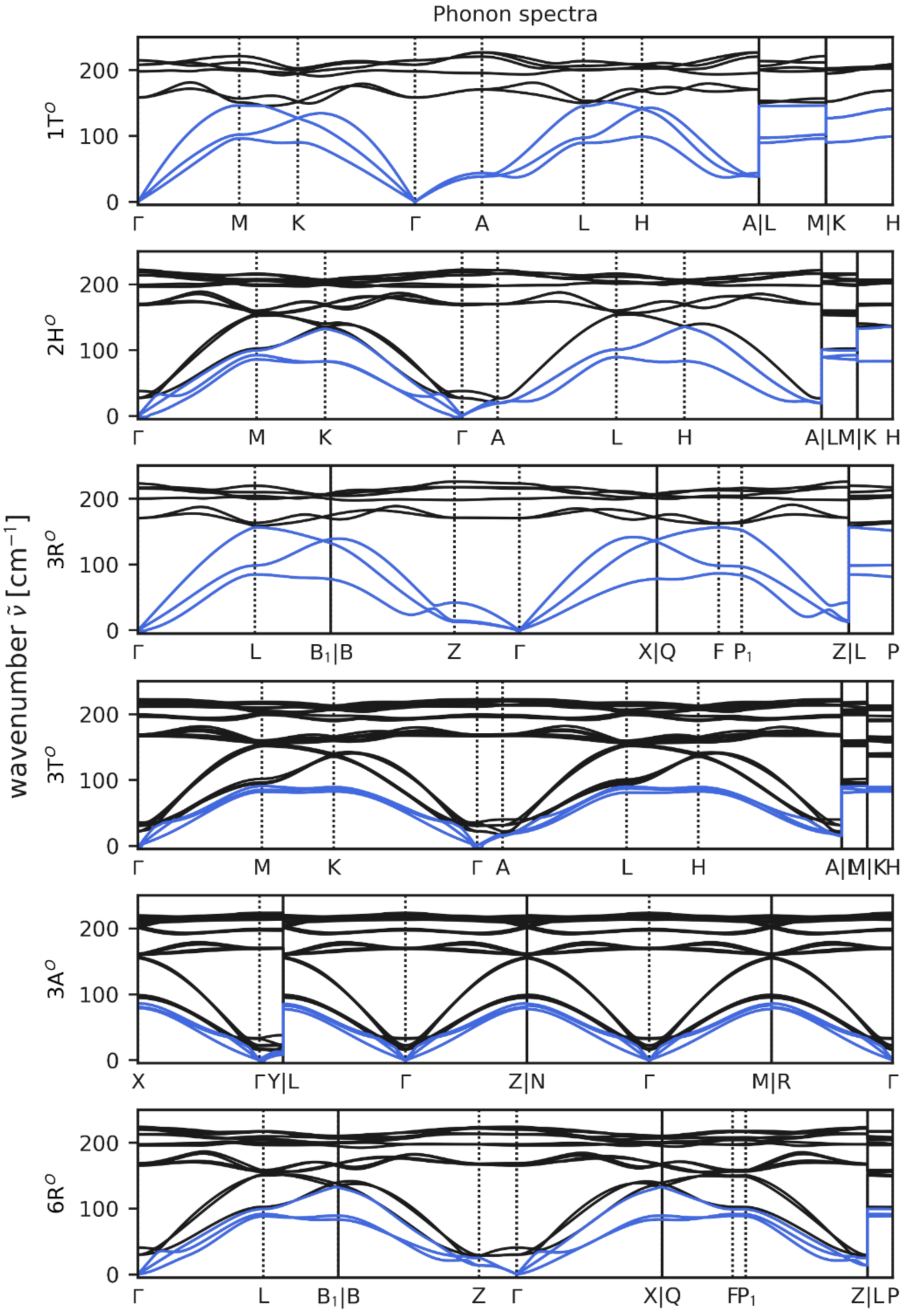

Figure S2 – Phonon spectra of six stacking phases of $PtSe_2$. The spectra show no imaginary frequencies and the structures are considered locally stable. Blue bands indicate the acoustic modes. The Brillouin zone paths follow the convention of Setyawan-Curtarolo.[1]

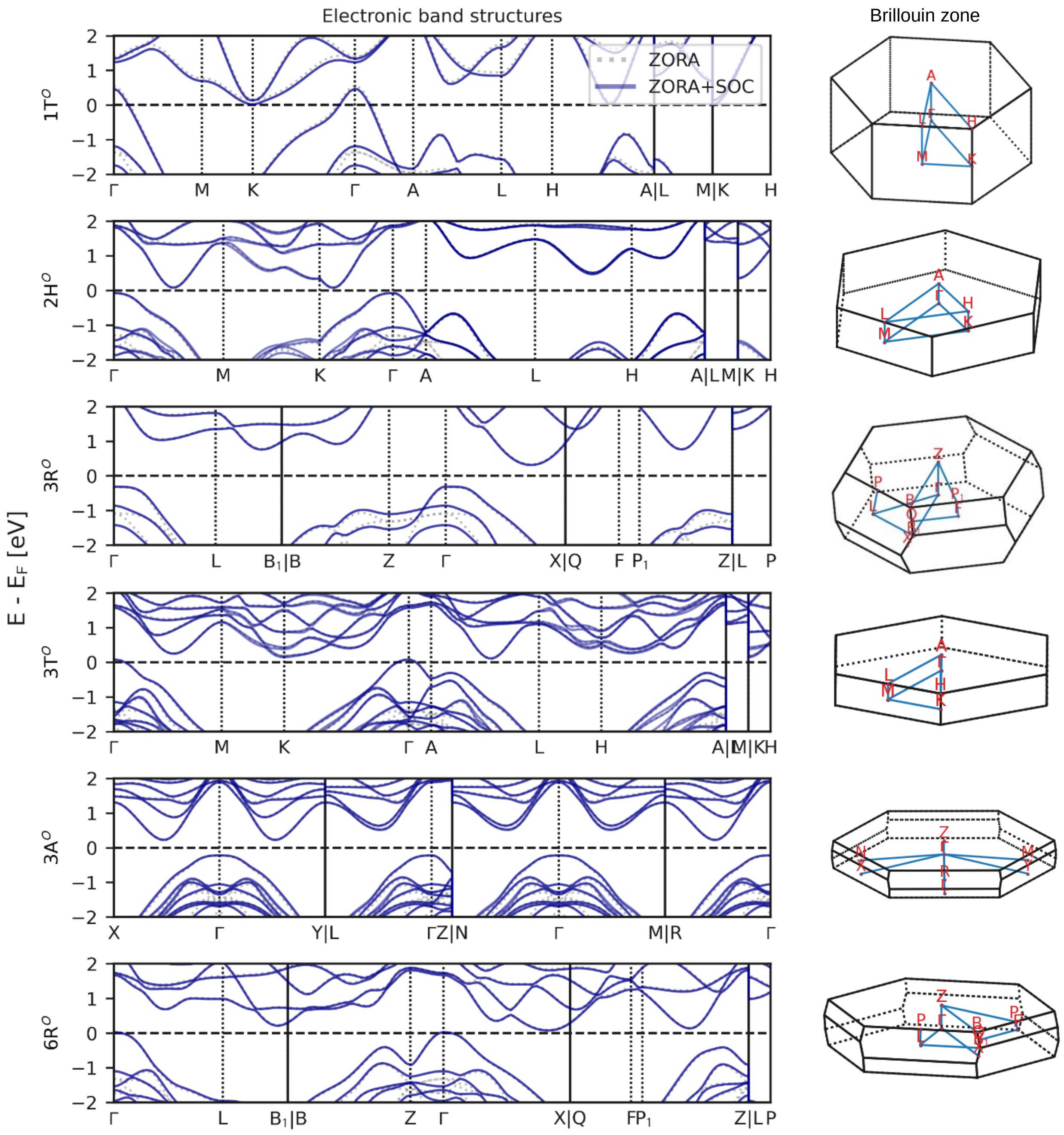

Figure S3 – Electronic band structures of six stacking phases of $PtSe_2$ at the HSE06 level of theory with and without spin-orbit coupling (SOC). The Brillouin zone paths follow the convention of Setyawan-Curtarolo.[1]

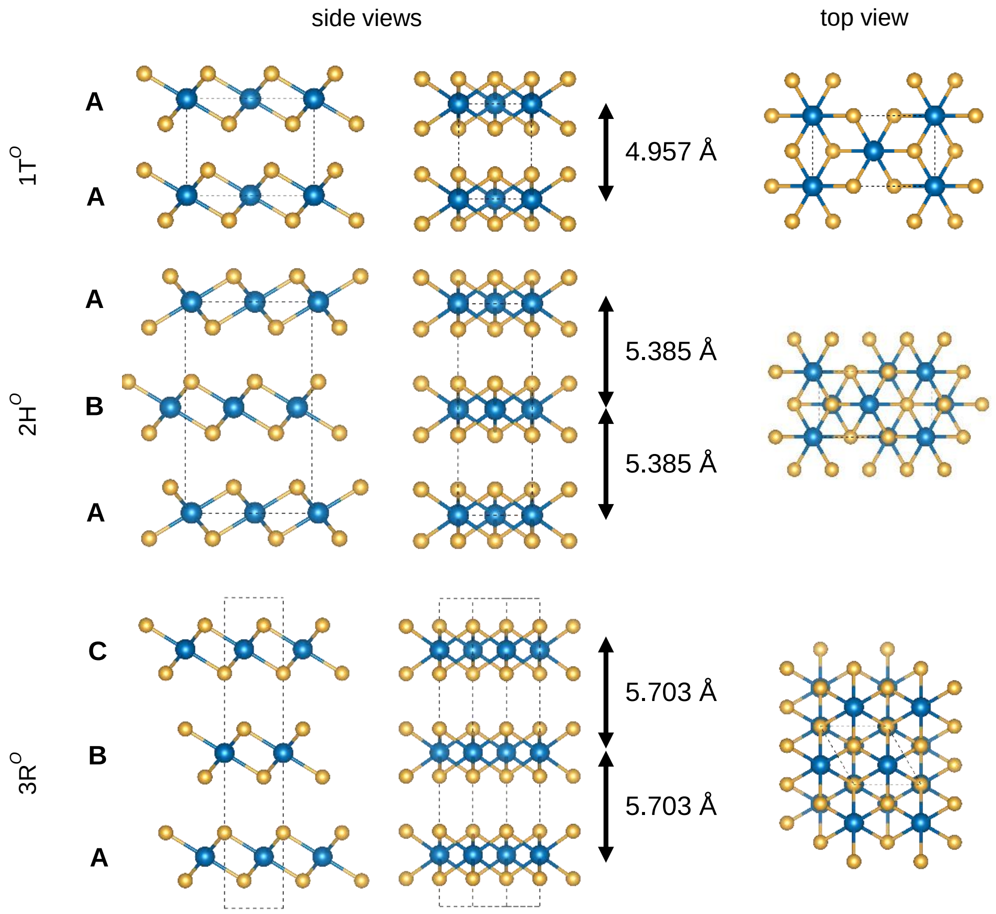

Figure S4 – Visualization of the $1T^{O}$, $2H^{O}$ and $3R^{O}$ stacking phases from different perspectives including stacking order and interlayer distance.

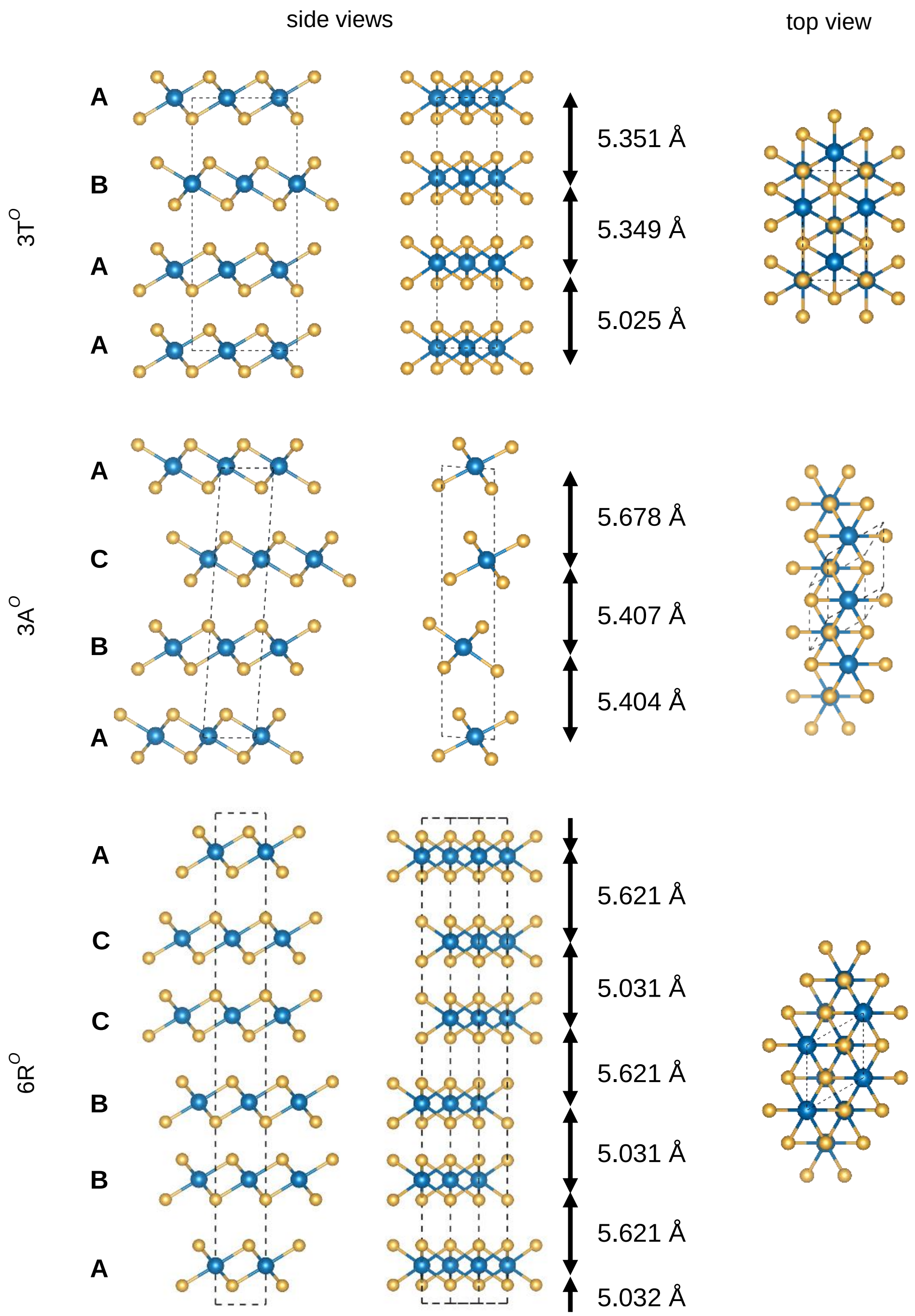

Figure S5 – Visualization of the $3R^O$, $3A^O$ and $6R^O$ stacking phases from different perspectives including stacking order and interlayer distance.

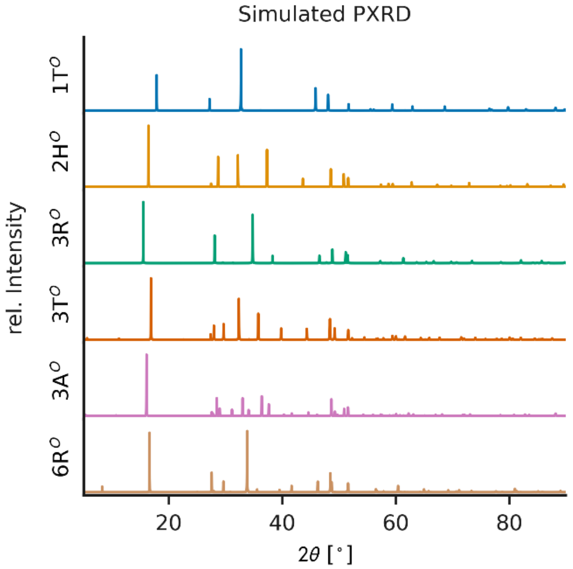

Figure S6 – Simulated powder x-ray diffraction (PXRD) pattern for the six bulk stacking phases with VESTA for a wavelength of $\lambda = 1.54059$ Å.

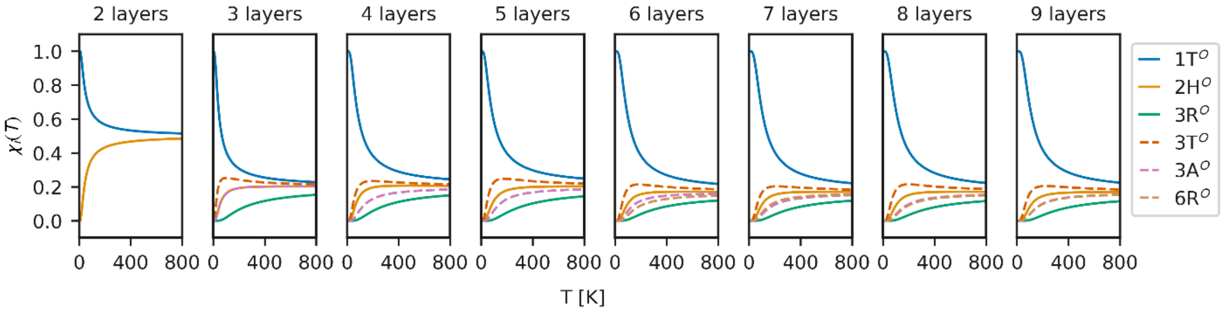

Figure S7 – Relative abundance vs. temperature for different layer numbers.

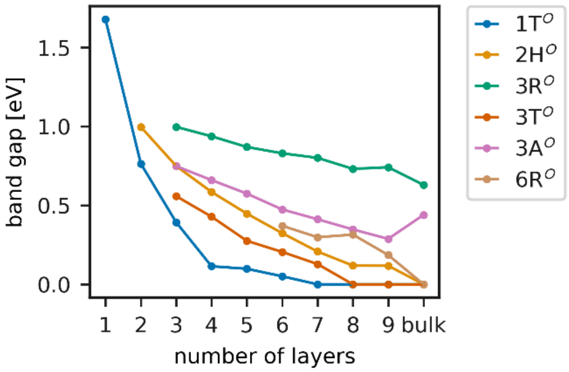

Figure S8 – Electronic band gap per layer number and stacking phase at the HSE06+SOC level of theory.

[1] A. H. Larsen, J. J. Mortensen, J. Blomqvist, I. E. Castelli, R. Christensen, M. Du\lak, J. Friis, M. N. Groves, B. Hammer, C. Hargus, E. D. Hermes, P. C. Jennings, P. B. Jensen, J. Kermode, J. R. Kitchin, E. L. Kolsbjerg, J. Kubal, K. Kaasbjerg, S. Lysgaard, J. B. Maronsson, T. Maxson, T. Olsen, L. Pastewka, A. Peterson, C. Rostgaard, J. Schiøtz, O. Schütt, M. Strange, K. S. Thygesen, T. Vegge, L. Vilhelmsen, M. Walter, Z. Zeng, K. W. Jacobsen, *J. Phys. Condens. Matter* **2017**, *29*, 273002.
[2] A. Togo, I. Tanaka, *ArXiv180801590 Cond-Mat* **2018**.
[3] V. Blum, R. Gehrke, F. Hanke, P. Havu, V. Havu, X. Ren, K. Reuter, M. Scheffler, *Comput. Phys. Commun.* **2009**, *180*, 2175.
[4] J. P. Perdew, K. Burke, M. Ernzerhof, *Phys. Rev. Lett.* **1996**, *77*, 3865.
[5] J. Hermann, A. Tkatchenko, *ArXiv191003073 Cond-Mat Physicsphysics* **2019**.
[6] F. Knoop, T. Purcell, M. Scheffler, C. Carbogno, *J. Open Source Softw.* **2020**, *5*, 2671.
[7] A. Togo, I. Tanaka, *Scr. Mater.* **2015**, *108*, 1.
[8] M. I. Aroyo, A. Kirov, C. Capillas, J. M. Perez-Mato, H. Wondratschek, *Acta Crystallogr. A* **2006**, *62*, 115.
[9] M. T. Dove, *Introduction to Lattice Dynamics*, Cambridge University Press, Cambridge ; New York, **1993**.
[10] J. Heyd, G. E. Scuseria, M. Ernzerhof, *J. Chem. Phys.* **2006**, *124*, 219906.
[11] Kermode, James, Pastewka, Lars, *Matscipy*, **n.d.**
[12] G. K. H. Madsen, J. Carrete, M. J. Verstraete, *Comput. Phys. Commun.* **2018**, *231*, 140.
[13] J. de Boor, E. Müller, *Rev. Sci. Instrum.* **2013**, *84*, 065102.
[14] R. Hill, *Proc. Phys. Soc. Sect. A* **1952**, *65*, 349.
[15] C. Yim, N. McEvoy, S. Riazimehr, D. S. Schneider, F. Gity, S. Monaghan, P. K. Hurley, M. C. Lemme, G. S. Duesberg, *Nano Lett.* **2018**, *18*, 1794.
[16] M. O'Brien, N. McEvoy, C. Motta, J.-Y. Zheng, N. C. Berner, J. Kotakoski, K. Elibol, T. J. Pennycook, J. C. Meyer, C. Yim, M. Abid, T. Hallam, J. F. Donegan, S. Sanvito, G. S. Duesberg, *2D Mater.* **2016**, *3*, 021004.
[17] S. Lukas, O. Hartwig, M. Prechtl, G. Capraro, J. Bolten, A. Meledin, J. Mayer, D. Neumaier, S. Kataria, G. S. Duesberg, M. C. Lemme, *Adv. Funct. Mater.* **2021**, 2102929.
